# Redirection of Cord Blood T Cells and Natural Killer Cells for Elimination of Autologous HIV-1-Infected Target Cells Using Bispecific DART® Molecules

**DOI:** 10.3389/fimmu.2020.00713

**Published:** 2020-04-21

**Authors:** Justin Pollara, R. Whitney Edwards, Shalini Jha, Chia-Ying Kao Lam, Liqin Liu, Gundo Diedrich, Jeffrey L. Nordstrom, Tori Huffman, Joy A. Pickeral, Thomas N. Denny, Sallie R. Permar, Guido Ferrari

**Affiliations:** ^1^Department of Surgery, Duke University School of Medicine, Durham, NC, United States; ^2^Human Vaccine Institute, Duke University School of Medicine, Durham, NC, United States; ^3^Macrogenics, Inc., Rockville, MD, United States; ^4^Department of Pediatrics, Duke University School of Medicine, Durham, NC, United States

**Keywords:** pediatric HIV-1, umbilical cord blood, cytotoxic T cells, natural killer cells, redirected cytotoxicity, bispecific DART molecules

## Abstract

Mother-to-child transmission of HIV-1 remains a major global health challenge. Currently, HIV-1-infected infants require strict lifelong adherence to antiretroviral therapy to prevent replication of virus from reservoirs of infected cells, and to halt progression of disease. There is a critical need for immune interventions that can be deployed shortly after infection to eliminate HIV-1-infected cells in order to promote long-term remission of viremia, or to potentially cure pediatric HIV-1-infection. Bispecific HIV × CD3 DART® molecules able to co-engage the HIV-1 envelope protein on the surface of infected cells and CD3 on cytolytic T cells have been previously shown to eliminate HIV-1 infected cells *in vitro* and are candidates for passive immunotherapy to reduce the virus reservoir. However, their potential utility as therapy for infant HIV-1 infection is unclear as the ability of these novel antibody-based molecules to work in concert with cells of the infant immune system had not been assessed. Here, we use human umbilical cord blood as a model of the naïve neonatal immune system to evaluate the ability of HIV x CD3 DART molecules to recruit and redirect neonatal effector cells for elimination of autologous CD4^+^ T cells infected with HIV-1 encoding an envelope gene sequenced from a mother-to-child transmission event. We found that HIV × CD3 DART molecules can redirect T cells present in cord blood for elimination of HIV-infected CD4^+^ T cells. However, we observed reduced killing by T cells isolated from cord blood when compared to cells isolated from adult peripheral blood—likely due to the absence of the memory and effector CD8^+^ T cells that are most cytolytic when redirected by bispecific DART molecules. We also found that newly developed HIV × CD16 DART molecules were able to recruit CD16-expressing natural killer cells from cord blood to eliminate HIV-infected cells, and the activity of cord blood natural killer cells could be substantially increased by priming with IL-15. Our results support continued development of HIV-specific DART molecules using relevant preclinical animal models to optimize strategies for effective use of this immune therapy to reduce HIV-1 infection in pediatric populations.

## Introduction

Despite the effectiveness of perinatal and postnatal antiretroviral prophylaxis, and the relatively low transmission rates of HIV-1 *in utero*, intrapartum, and during breast-feeding, there remain over 160,000 new pediatric HIV-1 infections annually worldwide (1–4). The overwhelming majority of these infections occur perinatally, via mother-to-child transmission. Treatment is critical as infant HIV-1 peak and set-point viral loads are typically 10-fold above those observed for primary adult infection and are highly predictive of disease outcome (5–12). With proper adherence, antiretroviral therapy (ART) can successfully control viremia in infants, and if initiated shortly after birth, ART may appreciably limit the size of the latently infected cell reservoir (13, 14). However, evidence to date suggests that strict adherence to ART will need to be maintained lifelong to prevent reemergence of virus replication resulting from reactivation of latent infection. Therefore, to provide long-term control or cure of pediatric HIV infection and abrogate the need for lifelong viral suppression, early initiation of ART will need to be combined with additional strategies to eliminate HIV-1-infected cells responsible for persistence of the virus reservoir.

One promising approach to increase elimination of HIV-infected cells is passive immunotherapy with antibody-based molecules capable of recruiting and redirecting endogenous cytolytic effector cells (15, 16). Bispecific HIV × CD3 DART® molecules based on HIV-specific monoclonal antibodies (mAbs) can effectively mediate *in vitro* elimination of HIV-1 infected and reactivated latently infected cells (17, 18). Bispecific HIV × CD3 DART molecules use a monovalent HIV-targeting arm comprised of the antigen-binding region of mAbs specific for highly conserved regions of the HIV envelope protein (Env) to recognize HIV-1-infected target cells, and a monovalent CD3 binding arm for recruitment of cytolytic T cells. Only when both arms are co-engaged will polyclonal T cells be activated and redirected for cytolytic responses against Env-expressing, HIV-1-infected target cells in a major histocompatibility complex-independent manner (18, 19). As a result of these properties, HIV × CD3 DART molecule-mediated activity should be unaffected by mutations among circulating or latent viruses that confer escape from viral-specific T-cell responses, or by the low frequency and functionality of HIV-specific T cells in patients on ART (20, 21). Therefore, passive immunization with HIV × CD3 DART molecules could form the basis of a strategy for cure of HIV by combining early initiation of ART to control viral load and reduce the size of the reservoir with concurrent initiation of HIV × CD3 DART molecule immunotherapy to eliminate infected cells. Once viral load is below detection, ART and DART molecule immunotherapy would be maintained with the addition of compounds that reactivate latent virus-infected cells to generate targets for DART molecule-mediated clearance.

Newborn infant infection resulting from mother-to-child transmission of HIV-1 (MTCT) likely represents the most favorable clinical context for successful passive immunotherapy to eliminate the reservoir of HIV-infected cells. Therapy can be initiated shortly after birth, and therefore near the time of transmission events occurring late *in utero*, during labor and delivery, or via breast milk —likely prior to the establishment of large populations of latently infected cells and the onset of immune dysregulation (22–25). However, neonatal cytolytic immune cells have phenotypic and functional differences compared to the same cell populations in adults (26, 27), and the impact of these differences on the activity of cytotoxic antibody-based immunotherapeutic drugs is not known. Thus, it is crucial to first evaluate infant passive immunotherapies in a model system that recapitulates the effector cells present in newborn infants.

In this study, we used human umbilical cord blood as surrogate for neonatal peripheral blood to test the hypothesis that bispecific DART molecules could redirect neonatal effector cells for elimination of cells infected with HIV-1 encoding an envelope gene sequenced from a mother-to-child transmission event. Our data demonstrate that HIV × CD3 DART molecules can redirect cord blood T cells to kill autologous cord blood CD4^+^ T cells infected by HIV-1 *in vitro*. However, we observed reduced killing by T cells isolated from cord blood when compared to those isolated from adult peripheral blood. We also found that HIV × CD16 DART molecules can recruit and redirect natural killer (NK) cells from cord blood to eliminate autologous HIV-infected CD4^+^ T cells; and we determined that the redirected activity of cord blood NK cells can be substantially increased by priming with IL-15. Our data suggest that HIV-specific DART molecules combined with current ART regimens may provide a novel treatment option intended to improve virus control, promote long-term remission, or cure pediatric HIV-1 infection. Our data also indicate that strategies to optimize and enhance the cytotoxic potential of neonatal effector cells, or allowing additional time after birth for the immune system to develop, will likely be needed to maximize the therapeutic potential of HIV-specific DART molecules for use in pediatric populations.

## Materials and Methods

### Study Samples

#### Blood and Mononuclear Cell Samples

Anonymized human umbilical cord blood donations that failed to meet the volume and/or nucleated cell count criteria required for clinical use were obtained with informed written consent. Human peripheral venous blood was collected by leukapheresis from healthy consenting adult volunteers (28, 29). All samples were collected in accordance with the policies and regulations of the Duke Health Institutional Review Board. Cord blood mononuclear cells (CBMC) and adult peripheral blood mononuclear cells (PBMC) were processed from umbilical cord blood and human peripheral blood, respectively, using density gradient centrifugation with Ficoll-Paque plus (GE Healthcare Life Sciences, Pittsburg, PA), and were cryopreserved in 10% DMSO 90% Fetal Bovine Serum (FBS). All samples were processed within 12 h after collection.

#### Bispecific HIV × CD3 and HIV × CD16 DART Molecules

HIV × CD3 DART molecules were constructed with anti-HIV-1 envelope specificities based on non-neutralizing mAbs, 7B2 or A32, or an irrelevant control specificity based on the anti-respiratory syncytial virus (RSV) mAb, palivizumab, and with an anti-human CD3ε specificity based on the mAb, hXR32, as described previously (18, 30). These molecules are referred to as 7B2 × CD3, A32 × CD3, and RSV × CD3. HIV × CD16 DART molecules were constructed with anti-human CD16 specificities based on mAb h3G8 as the effector arm, which included 7B2 × CD16 and 4420 × CD16, where 4420 is an anti-fluorescein (control) specificity, and Fc-bearing ones based on mAb h5H2 as the effector arm included 7B2 × CD16, A32 × CD16, and RSV × CD16. The human immunoglobulin G1 (IgG1) Fc domain, which is incorporated to prolong circulating half-life *in vivo*, contains mutations to greatly reduce or eliminate effector function via binding to Fc-gamma receptors (FcγRs) and complement, while retaining binding to the neonatal Fc receptor (FcRn) to take advantage of the IgG salvage pathway mediated by this receptor.

### Laboratory Methods

#### Phenotypic Characterization of T Cells

Immunophenotyping of human T cells was performed using flow cytometry analyses. Cryopreserved PBMC (5 donors) and CBMC (14 donors) were thawed and incubated overnight (18 h) in RPMI 1640 medium supplemented with 10% FBS at 37°C, 5% CO_2_. The cells were then washed with buffered saline and stained with a viability marker (Fixable Aqua Dead Cell Stain Kit, Thermo Fisher Scientific, San Diego, CA) prior to surface and intracellular staining with fluorescently conjugated antibodies using standard techniques as described (31). The staining panel used to identify T cell subsets and phenotypes was based on the Optimized Multicolor Immunofluorescence Panel 22 (OMIP-22) described previously (31). Fluorescently conjugated Abs used for cell surface staining were: APC-eFluor780-CD4 (clone RPA-T4, ebioscience, San Diego, CA); Horizon V500-CD14 (clone M5E2, BD Biosciences, San Jose, CA); Horizon V500-CD19 (clone HIB19, BD Biosciences); BV785-CD45RO (clone UCHL1, Biolegend, San Diego, CA); BV605-CCR7 (clone G043H7, Biolegend); and APC-CD366 (clone F38-2E2, Biolegend). Intracellular staining was performed with PE-TR-CD3 (clone UCHT1, Beckman Coulter, Brea, CA); and Ax700-CD8 (clone HIT8a, Biolegend). Data analyses were performed using FlowJo software (v10.5.3) from BD Biosciences.

#### Phenotypic Characterization of NK Cells and CD16 DART-Molecule NK Cell Binding

Immunophenotyping of human NK cells was performed as previously described (32). Cryopreserved PBMC collected from 15 healthy normal adult donors, and CBMC from 15 umbilical cord blood donors, were thawed and incubated overnight (18 h) in RPMI1640 medium supplemented with 10% FBS, or in RPMI 1640 medium supplemented with 10% FBS and 10 ng/mL recombinant human IL-15 (Miltenyi Biotec, GmbH) at 37°C, 5% CO_2_. The cells were then washed with buffered saline and stained with a viability marker (Fixable Aqua Dead Cell Stain Kit, Thermo Fisher Scientific) prior to surface and intracellular staining with fluorescently conjugated Abs using standard techniques as described previously (33). Fluorescently conjugated antibodies used for surface staining were based on OMIP-007 and are as follows: PE-TR-CD3 (clone S4.1, Thermo Fisher Scientific); APC-H7-CD4 (clone SK3, BD Biosciences); PE-Cy5-CD14 (clone Tuk4, Thermo Fisher Scientific); PE-Cy5-CD19 (clone SJ25-C1, Thermo Fisher Scientific); PacificBlue-CD16 (clone 3G8, BD Biosciences); PE-Cy7-CD56 (clone NCAM16.2, BD Biosciences); BV606-CD62L (clone DREG-56, Biolegend); FITC-HLA-DR (clone G46-6, BD Biosciences); APC-CD57 (clone HCD57, Biolegend); and BV785-CD69 (clone FN50, Biolegend). Intracellular staining was performed with BV711-Perforin (clone dG9, Biolegend), and PE-Granzyme B (clone GB11, BD Biosciences). Quantum™ Simply Cellular® beads (Bangs Laboratories, Inc., Fishers, Indiana) were used to determine the antibody binding capacity (ABC) of perforin and granzyme within cells according the manufacturers recommended procedure. Data analyses were performed using FlowJo software (v10.5.3).

To determine if DART molecules with CD16 targeting arms could specifically bind the surface of human NK cells, we incubated purified human NK cells with DART molecules at 1 μg/ml for 40 min at room temperature. Cells were then washed (PBS with 1% FBS) and incubated with 1 μg/ml mouse anti-EK antibody (recognizes the E/K heterodimerization region of DART molecules, MacroGenics) for 20 min at 4°C in wash buffer. Cells were then washed and incubated with PE rat-anti mouse IgG1 (BD Biosciences), at the manufactures recommended concentration, in wash buffer, for 25 min at 4°C. Finally, cells were washed, fixed, and analyzed by flow cytometry. Data are reported as mean fluorescent intensity (MFI) of PE.

#### Redirected T Cell Cytotoxicity Assay Against Autologous HIV-1 Env-Expressing CD4^+^ T Cells

Redirected T cell cytotoxicity assays were performed with methods similar to those previously described (18). Cryopreserved resting CBMC and PBMC from normal healthy donors were activated for 72 h with anti-human CD3 antibody (clone OKT3, eBioscience), anti-human CD28 antibody (clone 28.2, BD Biosciences) each at 150 ng/mL, and recombinant human IL-2 (30 U/mL, Proleukin, Prometheus Therapeutics and Diagnostics) in RPMI 1640 media supplemented with 20% FBS. Next, CD8^+^ T cells were depleted using magnetic beads (Miltenyi Biotec), leaving a CD4^+^ T cell-enriched population that was then infected with HIV-1 infectious molecular clone virus (HIV-1 4403bmC5) encoding a subtype C infant postnatally-transmitted/founder envelope protein (34) and Tat-inducible *Renilla* luciferase reporter gene (35), by spinoculation as described (36). Where indicated, cells were alternatively infected with an infectious molecular clone virus representing HIV-1 subtype B isolate BaL. After 48 h of infection, the CD4^+^ T cells were incubated with CD8^+^ T cells purified from autologous PBMC or CBMC using negative selection with magnetic beads (human CD8^+^ T cell isolation kit, Miltenyi Biotec) at a CD8^+^ T cell to target cell ratio of 30:1 in ½ area opaque flat bottom plates (Corning Life Sciences, Corning, NY). HIV × CD3 DART molecules were added, in duplicate, using 10-fold serial dilutions starting at 1 μg/mL, and the plates were incubated for an additional 24 h at 37°C, 5% CO_2_. Control plates included only infected CD4^+^ target cells and DART molecules without autologous CD8^+^ T effector cells. Percent of specific killing was calculated based on the change in Relative Light Units (RLU) (ViviRen luciferase assay; Promega, Fithchburg, WI) resulting from the loss of live, intact target cells in test wells containing effector cells, target cells, and DART molecules relative to RLU in control wells containing target cells and effector cells alone (without DART molecules) according to the following formula: percent of specific killing = [(number of RLU of control well – number of RLU of test well)/number of RLU of control well] ×100. Positive responses were defined as specific killing >20%. Data were fit to a sigmoidal dose-response function for graphing and for determination of 50% effective concentrations (EC_50_). In most cases, data are reported as positive area under the curve (pAUC), defined as the area under the log-transformed dilution curve that is above the threshold for positivity (20% specific killing), calculated with the trapezoidal method using SAS software (SAS Institute Inc., Cary, NC).

#### *In vitro* Evaluation of Strategies to Improve Redirected T Cell Cytotoxicity

Exogenous cytokines and T-cell co-stimulators were incorporated into the redirected T cell cytotoxicity assay to test their ability to improve the cytolytic activity of T cells present in CBMC and PBMC. The impact of exogenous IL-12 was evaluated by overnight incubation of the CBMC and PBMC with exogenous recombinant human IL-12 (10 ng/mL, Miltenyi Biotec), before isolation of CD8^+^ T cells used as effector cells in the redirected cytotoxicity assay. IL-12 was maintained in the assay plates at 10 ng/mL throughout the 24-h killing assay. The impact of coreceptor signaling was assessed by incubating the CBMC and PBMC overnight with anti-CD28 (150 ng/mL, clone 28.2, BD Biosciences) or anti-CD137 (150 ng/mL, clone 4B4-1, BioLegend), prior to isolation of CD8^+^ T cells, and maintaining the presence of the anti-coreceptor antibodies throughout the killing assay. Experiments were also performed using exogenous IL-12, anti-CD28, and anti-CD137 together, or with cells stimulated for 48 h by incubation with 150 ng/mL anti-human CD3 antibody (clone OKT3, eBioscience), anti-human CD28 antibody, and 30 U/mL recombinant human IL-2 in RPMI 1640 media supplemented with 20% FBS prior to isolation of CD8^+^ T cells. In one set of experiments, T cells in CBMC and PBMC were activated for 72 h with anti-CD3, anti-CD28, and IL-2, before isolation of CD8^+^ T cells, which were then allowed to rest for 72 h in absence of any stimulation before use as effector cells according to the methods described above.

#### Redirected NK Cell Cytotoxicity Assay Against Autologous HIV-1 Env-Expressing CD4^+^ T Cells

Cryopreserved CBMC and PBMC samples were thawed and rested overnight (18 h) at 37°C 5% CO_2_ in RPMI 1640 media supplemented with 10% FBS, or media supplemented with 10 ng/mL recombinant human IL-15 (Miltenyi Biotec), as previously described (32). Subsequently, NK cells were purified by negative selection with magnetic beads (human NK cell isolation kit, Miltenyi Biotec) and used as effector cells (NK cell to target cell ratio of 5:1) in redirected cytotoxicity assays using HIV × CD16 DART molecules, performed as described above. Positive responses were defined as specific killing >20%, and data were not fit to a dose-response function for graphing due to the presence of a prozone (37).

#### CD107a Degranulation Assay

Cell-surface expression of CD107a was used as a marker for T cell degranulation (38). Redirected cytotoxicity assays were performed as described above, with the following modification [as previously described in (18)]. After plating the target cells, effector cells, and DART molecules, the plate was incubated for 18 h and then FITC- or APC-Cy7-CD107a antibody (clone H4A3, BD Biosciences), brefeldin A (GolgiPlug, 1 μL/mL, BD Biosciences), and monensin (GolgiStop, 4 μL/6mL, BD Biosciences) were added to each well and the plates were incubated for an additional 6 h. Plates were then washed and stained with a viability marker and fluorescently conjugated antibodies as described above (phenotypic characterization of T cells), or with a truncated staining panel that included only antibodies specific for CD3, CD4, and CD8, using standard techniques. Data analyses were performed using FlowJo software (v10.5.3).

#### Statistical Methods

Kruskal Wallis tests were used to compare response magnitudes across groups. In order to assess if two groups have different response magnitudes, pairwise comparisons between groups were conducted using Wilcoxon rank sum test. Two-tailed *p* < 0.05 were considered significant. Statistical analysis was performed using SAS software (SAS Institute Inc.).

## Results

### Cord Blood T Cells Have Modest Ability to Eliminate Autologous HIV-Infected CD4 T Cells Directed by HIV x CD3 DART Molecules *in vitro*

**Figure 1:**
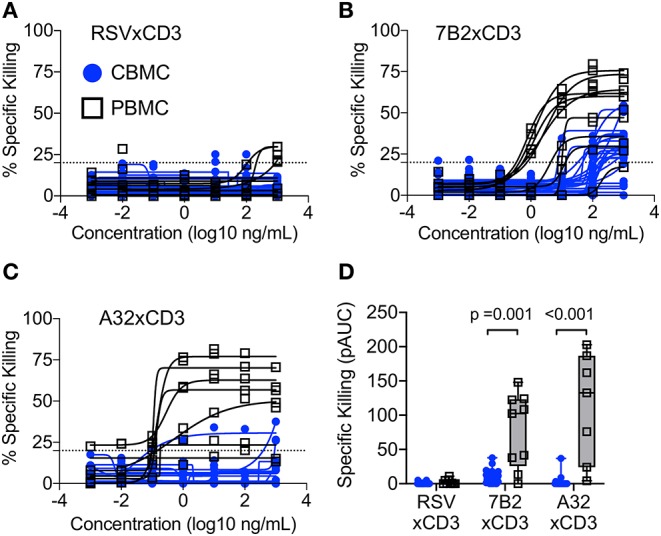
Redirection of cytolytic T cells to autologous HIV-infected CD4^+^ T cells by HIV × CD3 DART molecules. The ability of T cells isolated from cord blood mononuclear cells (CBMC, blue lines, and symbols) and adult peripheral blood mononuclear cells (PBMC, black lines, and open symbols) to eliminate autologous CD4^+^ T cells infected with an HIV-1 molecular clone virus representing an infant postnatally-transmitted founder isolate (HIV-1 4403bmC5) after being redirected by **(A)** RSV × CD3 control DART molecules (*n* = 25 CBMC samples, *n* = 9 PBMC samples), **(B)** 7B2 × CD3 DART molecules (*n* = 25 CBMC samples, *n* = 9 PBMC samples), and **(C)** A32 × CD3 DART molecules (*n* = 18 CBMC samples, *n* = 7 PBMC samples). Dashed line indicates the threshold for positivity. The positive areas under the dilution curves (pAUC) are presented in **(D)** with blue circles and bars representing assays performed with cells from CBMC and black/gray squares and bars representing assays performed with cells from adult PBMC. Box plots represent the interquartile ranges, horizontal lines indicate the medians, and error bars extend to the minimum and maximum observed values. Data are from 24 h killing assays with a CD8^+^ effector to HIV-infected autologous CD4^+^ target cell ratio of 30:1.

We and others have demonstrated that bispecific DART molecules able to co-engage HIV-1 Env on infected cells, and CD3 antigen on cytolytic T cells, can be used to eradicate acutely infected and reactivated latently-infected cells *in vitro* (17, 18). However, these initial studies were performed using immune cells from adults —the ability of these novel antibody-based molecules to work in concert with cells present in the neonatal immune system cells had not been explored. To address this limitation, we performed redirected T cell cytotoxicity assays using CBMC as a source of neonatal CD8^+^ T cells for use as effectors, and autologous cord blood CD4^+^ T cells for use as targets after infection with HIV-1 *in vitro*. Target cells were infected with a subtype C HIV-1 infectious molecular clone virus that encodes the *env* gene from HIV-1 4403bmC5, a postnatally-transmitted founder virus isolated from infant plasma, and identical to a virus isolate present in matched maternal breast milk (34). Thus, this isolate is expected to be a representative of those present in the early active and latent virus reservoirs of infected infants. Assays were also performed using adult PBMC as a source of effector cells and autologous HIV-1 4403bmC5-infected target cells for comparison. We observed minimal background activity (specific killing <20%) by the negative control DART molecule, RSV × CD3, in assays performed with CD8^+^ T cells and autologous 4403bmC5-infected CD4^+^ cells isolated from adult PBMC (black lines and open squares, Figure 1A) or CBMC (blue lines and circles, Figure 1A). In contrast, HIV-specific DART molecules, 7B2 × CD3 and A32 × CD3, mediated specific killing activity with cells isolated from most donors (80% of CBMC samples, and 89% of PBMC samples with 7B2 × CD3; 17% of CBMC and 100% of PBMC samples with A32 × CD3) as shown in Figures 1B,C, respectively. However, we observed that the specific killing activity of cells from cord blood is reduced when compared to those from adult PBMC for the majority of samples, both in terms of maximum observed killing activity (Figures 1B,C), and EC_50_ (mean ~100 ng/mL for 7B2 × CD3 with CBMC samples that were positive for killing, and ~4 ng/mL with positive PBMC samples). To better compare the killing activities, we calculated the positive area under the dilution curves (Figure 1D), and confirmed that specific killing of HIV-1 4403bmC5-infected cells was significantly lower in assays performed with cells isolated from CBMC compared to adult PBMC (*p* ≤ 0.001, Wilcoxon rank sum tests). These data demonstrate that HIV-specific DART molecules can redirect cord blood T cells to kill HIV-1 infected, Env-expressing, autologous cord blood CD4^+^ T cells, however with activity subordinate to that mediated by adult peripheral blood T cells. Therefore, we next sought to identify characteristics of cord blood T cells that may limit their functionality when redirected by HIV-specific DART molecules.

### Comparison of Effector and Memory T Cell Subsets in Cord Blood and Adult Peripheral Blood

**Figure 2:**
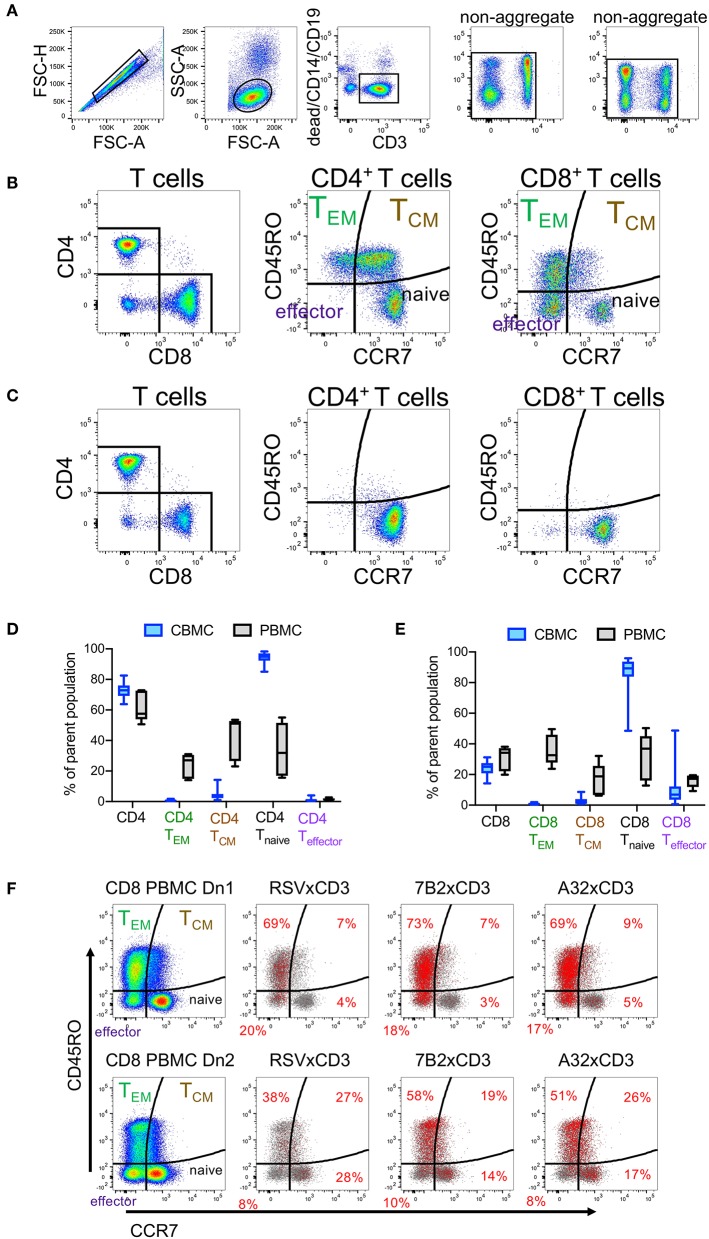
Comparison of T cell subsets in adult peripheral blood mononuclear cells (PBMC) and cord blood mononuclear cells (CBMC) **(A)** Gating strategy. **(B)** Flow cytometry plots showing representative example of CD4^+^ and CD8^+^ T cell subsets among adult PBMC or **(C)** CBMC. Comparison of frequencies of **(D)** total CD4^+^ T Cells and CD4^+^ T cell subsets or **(E)** CD8^+^ T cells and CD8^+^ T cell subsets in CBMC (*n* = 14) and adult PBMC (*n* = 5). In **(D,E)**, box plots represent the interquartile ranges, horizontal lines indicate the medians, and error bars extend to the minimum and maximum observed values. **(F)** CD8^+^ T cells subsets that degranulate concomitant with HIV × CD3 or control (RSV × CD3) DART molecule mediated cytolytic activity. Rows represent assay results using PBMC from two different adult donors, Dn1 and Dn2. Distribution of CD8^+^ T cell subsets is shown in the first column. Red dots in the remaining columns represent CD8 T cells that have degranulated (CD107a+) in response to the indicated DART molecules (frequencies within each quadrant indicated with red text), overlaid on the total CD8^+^ T cells (gray dots) acquired for each condition. 24 h killing assay with a CD8^+^ effector to Bal-infected autologous CD4^+^ target cell ratio of 30:1 and DART molecule concentration of 100 ng/mL.

We used flow cytometry immunophenotyping to compare T cell subsets present in CBMC to those present in adult PBMC. Comparisons were made using cells isolated from 14 cord blood and 5 adult peripheral blood samples. The gating strategy used to identify total T cells, and T cell subsets —naïve, effector, effector memory (T_EM_), and central memory (T_CM_)— is indicated in Figure 2A. Flow plots showing representative examples of CD4^+^ and CD8^+^ T cell subsets among PBMC and CBMC are shown in Figures 2B,C, respectively. Consistent with prior observations (39, 40), we found that although CBMC contained similar frequencies of CD4^+^ and CD8^+^ T cells as adult PBMC, there were marked differences in the distribution of functional subsets with cord blood CD4^+^ (Figure 2D) and CD8^+^ T cells (Figure 2E) being predominantly naïve (naïve CD4: median 95%, interquartile range 92–97%; naïve CD8: median 89%, interquartile range 84–94%), and therefore deficient in memory and effector subsets. We hypothesized that although DART molecules are able to redirect resting T cells for killing of HIV-infected cells without need for pre-activation (17, 18), they may be more effective when redirecting effector or memory subsets. To test this, we assessed CD107a degranulation concomitant with redirected killing activity with PBMC from two healthy adult donors as a source of effector cells, and HIV-1 subtype B BaL IMC virus-infected cells as targets. We previously demonstrated high levels of HIV × CD3 DART molecule-induced CD8^+^ T cell degranulation in the presence of CD4^+^ cells infected by this HIV-1 isolate (18). We found that although HIV-specific DART molecules induced degranulation in all subsets of CD8^+^ T cells during redirected cytotoxicity assays, the majority of degranulated cells were CCR7^−^ and therefore expressed phenotypes consistent with T_EM_ and effector cells (Figure 2F). These data suggest that the reduced DART molecule-mediated cytotoxicity observed in assays performed using CBMC-derived T cells is likely due in part to the predominance of naïve cells in cord blood, and the absence of memory and effector CD8^+^ T cells, which are preferentially redirected by the HIV × CD3 DART molecules.

### Evaluation of Strategies to Increase the Cytolytic Potential of T Cells in Cord Blood

**Figure 3:**
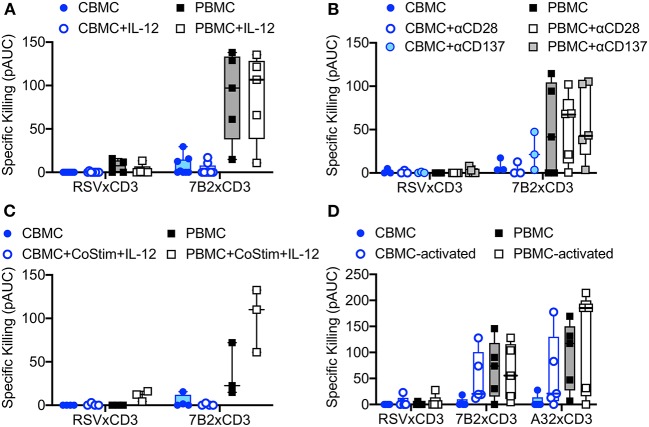
Redirection of cord blood mononuclear cell (CBMC)-derived cytolytic T cells for elimination of autologous HIV-1 infected CD4 T cells by HIV × CD3 DART molecules is not improved by incubation with **(A)** 10 ng/mL exogenous IL-12 (CBMC *n* = 8, PBMC *n* = 5), **(B)** costimulation with anti-CD28 or anti-CD137 antibodies (CBMC *n* = 3, PBMC *n* = 5), or **(C)** a combination of costimulation with anti-CD28 and anti-CD137 (CoStim) and exogenous IL-12 (CBMC *n* = 4, PBMC *n* = 3). **(D)** HIV × CD3 DART molecule-mediated elimination of autologous HIV-1-infected CD4^+^ T cells is improved when CD8^+^ T cells used as effectors are activated by T cell receptor stimulation (anti-CD28, anti-CD3, and IL-2) for 48 h prior to use in the cell killing assays (CBMC and PBMC *n* = 5). Box plots represent the interquartile ranges, horizontal lines indicate the medians, and error bars extend to the minimum and maximum observed values. 24 h killing assays with a CD8^+^ effector to HIV-1 4403bmC5-infected autologous CD4^+^ target cell ratio of 30:1. All data reported as positive area under the dilution curve (pAUC).

We explored strategies to increase the activity of cord blood cytolytic T cells for HIV DART molecule-redirected elimination of HIV-infected cells. We first evaluated the impact of priming CBMC-derived T cells with IL-12, as this cytotoxic-T-lymphocyte-promoting cytokine is known to be poorly expressed by the naïve infant immune system (41). We found that overnight incubation with exogenous IL-12, and maintenance of IL-12 treatment throughout the redirected cytotoxicity assay, did not improve cytotoxic activity of cord blood CD8^+^ T cells when used in combination with HIV × CD3 DART molecules (Figure 3A), nor was there any impact on activity of CD8^+^ T cells from adult PBMC. Next, reasoning that the naïve CD8^+^ T cells present in cord blood cells may require a more substantial activation signal compared to CD8^+^ T cells present in adult PBMC, we explored if killing activity could be improved by providing additional co-stimulatory signaling via co-engagement of CD28 or CD137 receptors (42). However, the results of experiments performed using T cells isolated from CBMC and adult PBMC demonstrated that exogenous anti-CD28 or anti-CD137 co-stimulation did not significantly improve killing mediated by HIV × CD3 DART molecules (Figure 3B). Moreover, we found that treatment of CBMC with IL-12 combined with anti-CD28 and anti-CD137 co-stimulation also had no impact on the cytolytic activity of T cells from CBMC when used with HIV × CD3 DART molecules, but did increase activity in assays performed with adult PBMC (Figure 3C), although the increase did not reach statistical significance (*p* = 0.064, Wilcoxon rank sum test). We next assessed if pre-activation of cord blood T cells would increase their cytotoxic activity. Interestingly, we found that pre-activation of cord blood T cells via T cell receptor (TCR) cross linking using anti-CD3 and anti-CD28 monoclonal antibodies in combination with exogenous recombinant human IL-2 (30 U/mL) improved the ability of T cells from 5 of 5 tested cord blood samples to eliminate HIV-infected cells upon redirection by 7B2 × CD3 DART molecules, and 4 of 5 tested samples upon redirection by A32 × CD3 DART molecules (Figure 3D). This observation indicates that CD8^+^ T cells present in cord blood are capable of improved cytotoxic activity when given sufficient stimulation.

**Figure 4:**
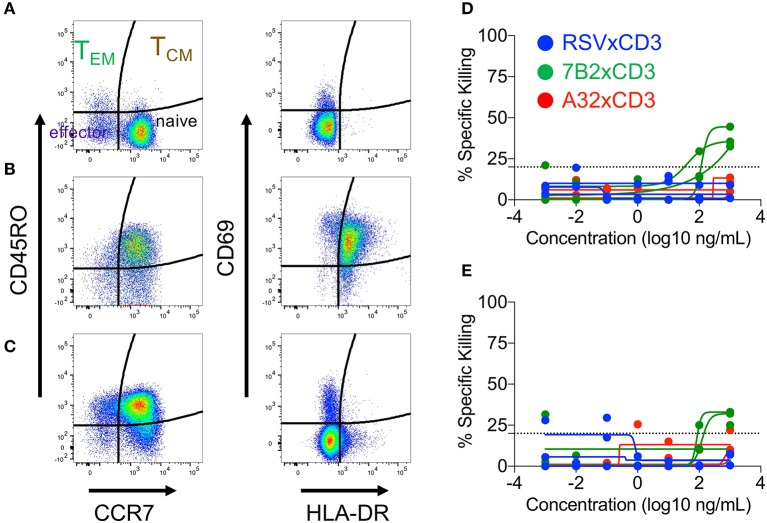
Cord blood mononuclear cell (CBMC) CD8^+^ T cells subset distribution (left panels) and activation state (right panels) after **(A)** overnight rest, **(B)** 3 days of T cell receptor stimulation (anti-CD3, anti-CD28, and IL-2), or **(C)** 3 day transition from activated cells back to the resting state. Representative example shown. Similar levels of DART molecule-redirected killing of autologous HIV-1 4403bmC5-infeced CD4^+^ T cells were observed in assays performed with **(D)** CD8^+^ T cell effectors after overnight rest, and **(E)** as they returned back to a resting state following 3 days of T cell receptor stimulation. **(D,E)** represent data from 24 h killing assays with a CD8^+^ effector to HIV-1 4403bmC5-infected autologous CD4^+^ target cell ratio of 30:1, performed using three different CBMC donors, each represented by individual lines.

We next determined if cord blood-derived CD8^+^ cells, which had experienced prior activation and subsequently transitioned back to a resting state, were able to maintain higher cytolytic activity. To test this, we activated cord blood CD8^+^ T cells for 72 h via TCR stimulation as described above, then allowed the cells to rest for an additional 72 h before use in the redirected killing assay. Flow cytometry analysis was used to define cell phenotypes during activation, and the return to resting state as shown in Figures 4A–C. As previously described, the preponderance of resting cord blood CD8^+^ T cell presented a naïve (CD45RO^−^, CCR7^+^) cell surface phenotype (Figure 4A). CD45RO expression increased along with expression of the activation markers CD69 and HLA-DR after TCR stimulation (Figure 4B). Upon returning to a resting state, cell-surface expression of activation markers CD69 and HLA-DR were reduced, but the vast majority of cells maintained a cell-surface phenotype consistent with that of T_CM_ cells (CD45RO^+^, CCR7^+^, Figure 4C). Despite these changes to cell-surface phenotypes, induced during activation and maintained after returning to rest, we observed similarly low levels of cytolytic activity for resting cord blood CD8^+^ T cells (Figure 4D), and CD8^+^ T cells that were transitioned from an active to resting state *in vitro* (Figure 4E). Collectively, these results demonstrated that redirected killing activity of cord blood T cells mediated by HIV × CD3 DART molecules was not affected by providing exogenous IL-12 or costimulatory signals, but could be improved when given sufficient stimulation leading to activation. However, the improved cytolytic activity was not maintained when activation waned.

### Cord Blood CD4^+^ T Cells Are Recruited by HIV × CD3 DART Molecules

**Figure 5:**
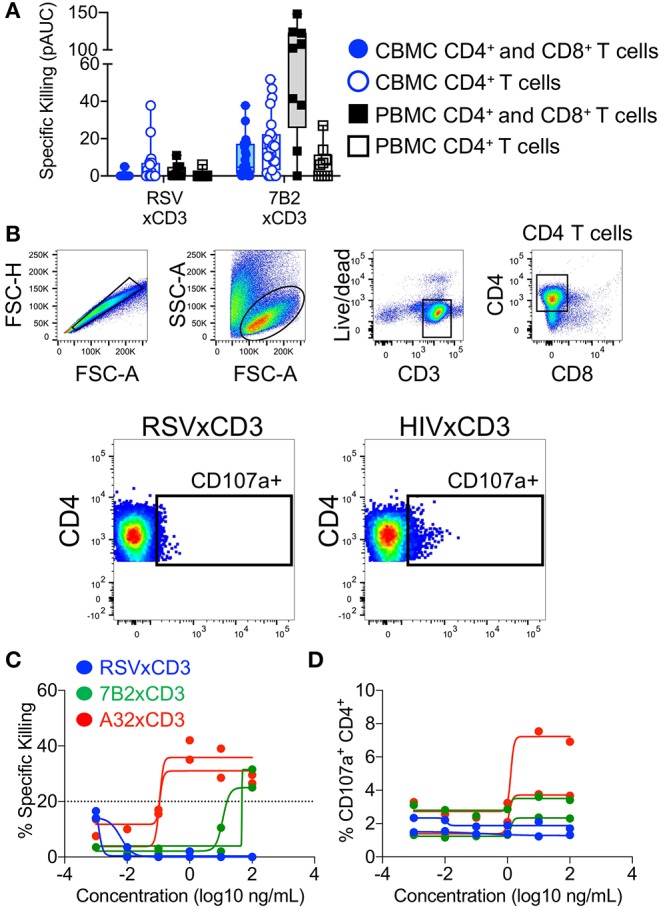
Redirection of cord blood mononuclear cell (CBMC)-derived CD4^+^ T cells for elimination of HIV-infected cells by HIV × CD3 or control (RSV × CD3) DART molecules. **(A)** Similar levels of HIV-1 4403bmC5 infected CBMC-derived CD4^+^ T cell killing were observed in assays performed with autologous CD8^+^ T cell effectors (filled blue circles and box, *n* = 23 CBMC samples) or with CD4^+^ T cells alone, no additional effector cells (open blue circles and box). HIV-1 4403bmC5 infected PBMC-derived CD4^+^ T cell killing observed in assays performed with autologous CD8^+^ T cell effectors (filled black squares and box, *n* = 9 PBMC samples) or with CD4^+^ T cells alone, no additional effector cells (open black squares and box). **(B)** Gating strategy used to identify cord blood CD4^+^ T cells that degranulated concomitant with DART molecule mediated cytolytic activity. Redirection of CBMC-derived CD4^+^ T cells for elimination of HIV-1 Bal-infected autologous CD4^+^ T cells as measured by **(C)** luciferase cell killing assay, and **(D)** CD107a degranulation assay. Data are from 24 h killing assays performed using two different CBMC donors, each represented by individual lines.

In parallel to the assays performed using CBMC-derived CD8^+^ T cells as effector cells, we also evaluated whether HIV × CD3 DART molecules had anti-HIV activity when incubated with infected cord blood CD4^+^ T cells alone. As the epitope recognized by the 7B2 × CD3 DART molecules is non-neutralizing (43) we expected minimal anti-HIV activity in absence of CD8^+^ effector cells. In contrast, we observed 7B2 × CD3 DART molecule-dependent specific killing of HIV-infected cells at similar levels for assays performed with and without CD8^+^ T cells from CBMC (Figure 5A, filled and open blue circles, respectively). We also observed 7B2 × CD3 DART molecule-dependent specific killing in assays performed without CD8^+^ T cells from PBMC (Figure 5A, open black squares), however killing was higher in the presence of PBMC-derived CD8^+^ T cells (Figure 5A, filled black squares). To determine if HIV × CD3 DART molecules were capable of activating effector responses from the cord blood CD4^+^ T cells themselves, we assessed CD107a degranulation during redirected cytotoxicity assays as described previously. The gating strategy for identification of cord blood-derived CD4^+^ T cells in this assay is shown in Figure 5B, with representative flow cytometry plots depicting detection of CD107a on CD4^+^ T cells in presence of control or HIV × CD3 DART molecules. These data demonstrate that CD4^+^ T cells from CBMC are capable of being induced to degranulate by DART molecules in redirected cytotoxicity assays. The specific killing activity observed using cord blood CD4^+^ T cells as effectors to eliminate HIV-1 BaL-infected autologous T cells from two cord blood donations is shown in Figure 5C. The frequency of CD107a^+^ CD4^+^ T cells detected in these assays is shown in Figure 5D. The specific killing and concomitant degranulation responses observed across the tested HIV × CD3 DART molecule concentrations show comparable trends, suggesting that degranulating CD4^+^ T cells contribute to the *in vitro* elimination of HIV-1 infected cells when redirected by HIV × CD3 DART molecules. This finding is consistent with observations made in our prior studies performed using adult PBMC (18).

### HIV × CD16 DART Molecules Can Recruit Cord Blood NK Cells for Elimination of Autologous HIV-1 Infected CD4^+^ T Cells

**Figure 6:**
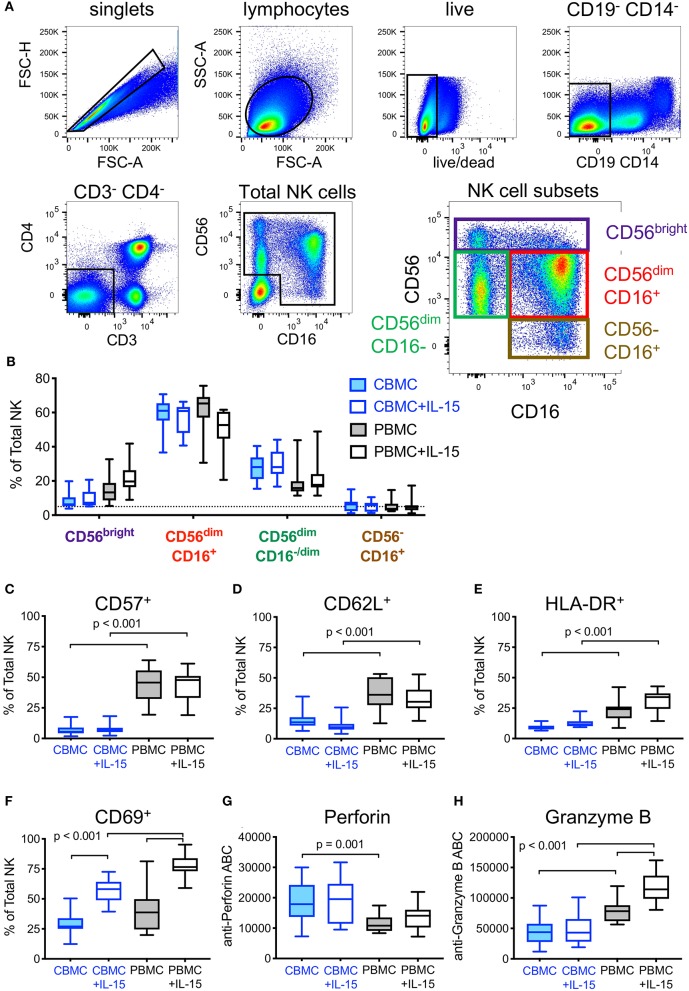
Effect of exogenous IL-15 treatment on phenotypes of natural killer (NK) cells present in cord blood mononuclear cells (CBMC) with comparison to adult peripheral blood (PBMC). **(A)** Gating strategy used to identify total and subsets of NK cells. Data representing all CBMC samples (*n* = 30, 15 CBMC donors +/- IL-15) were combined and included in the flow cytometry plots shown. **(B)** Comparison of NK cell subset frequencies between untreated, and after overnight treatment with 10 ng/mL IL-15 (*n* = 15 CBMC and *n* = 15 PBMC samples per condition). Frequencies of total NK cells expressing **(C)** CD57, **(D)** CD62L, **(E)**, HLA-DR, and **(F)** CD69 in cells left untreated or treated overnight with 10 ng/mL IL-15. Amount of intracellular **(G)** Perforin and **(H)** Granzyme B in NK cells left untreated or after overnight treatment with 10 ng/mL IL-15, measured by antibody binding capacity (see Methods). In **(B–H)**, box plots represent the interquartile ranges, horizontal lines indicate the medians, and error bars extend to the minimum and maximum observed values.

Another approach for increasing HIV DART-molecule-mediated elimination of HIV-1 infected cells is to recruit additional populations of cytotoxic effector cells. We used flow cytometry immunophenotyping to compare NK cells present in CBMC and adult PBMC according to the gating strategy shown in Figure 6A. We found that cord blood contains more total NK cells (median 11% total NK in CBMC and 5% in adult PBMC), but similar distributions of NK cell subsets, as adult peripheral blood (Figure 6B). As we, and others, have previously demonstrated that pretreatment with IL-15 augments the cytotoxic potential of NK cells (32, 44–46) we compared the impact of overnight incubation with IL-15 (10 ng/mL) on the subset distribution, activation state, and intracellular abundance of cytotoxic effector molecules in NK cells present in CBMC and adult PBMC. We previously showed that IL-15 treatment increased the frequency of CD56^bright^ NK cells and CD56^dim^CD16^−/dim^ NK cells, and decreased CD56^dim^CD16^+^ NK cells in adult PBMC [(32) and Figure 6B]. The impact of IL-15 on cord blood NK cell subset distributions was modest, with no significant change (Wilcoxon rank sum tests) in the frequencies of major NK cell subsets (Figure 6B). Importantly, the cytotoxic CD56^dim^CD16^+^ NK cell subset was the dominant population of NK cells in both cord blood and adult PBMC. As we previously described for IL-15 treated NK cells in adult PBMC, we found that IL-15 treatment did not impact the expression of the maturation marker CD57 on the surface of cord blood NK cells, although its expression was markedly lower than that observed on NK cells in adult PBMC (*p* < 0.001, Wilcoxon rank sum test), which is consistent with the naivety of cord blood immune cells (Figure 6C). We also found that fewer cord blood NK cells expressed the secondary lymphoid homing marker CD62L when compared to NK cells in adult PBMC (*p* < 0.001, Wilcoxon rank sum test), and treatment of cord blood with IL-15 further reduced the percentage of NK cells expressing CD62L (Figure 6D). Moreover, and as expected, cord blood NK cells incubated with IL-15 presented a more active phenotype than untreated NK cells as evident by cell surface expression of HLA-DR (Figure 6E) and CD69 (Figure 6F), although overall, the frequency of activated NK cells was lower in CBMC compared to adult PBMC following IL-15 treatment (*p* < 0.001 for both CD69 and HLA-DR, Wilcoxon rank sum tests). Finally, using fluorescent quantitation beads to measure the intracellular content of perforin and granzyme B, we found that cord blood NK cells contained more perforin (Figure 6G) but less granzyme B (Figure 6H) than NK cells in adult PBMC, whether untreated (perforin, *p* = 0.001; granzyme B, *p* < 0.001, Wilcoxon rank sum tests) or treated with IL-15 (granzyme B, *p* < 0.001, Wilcoxon rank sum test). Collectively, these data suggest that cord blood contains similar distributions of NK cell subsets as adult peripheral blood, however the NK cells present in cord blood are less mature, less lymphoid homing, less active, and contain less of the cytotoxic effector molecule granzyme B when compared to NK cells in adult PBMC.

**Figure 7:**
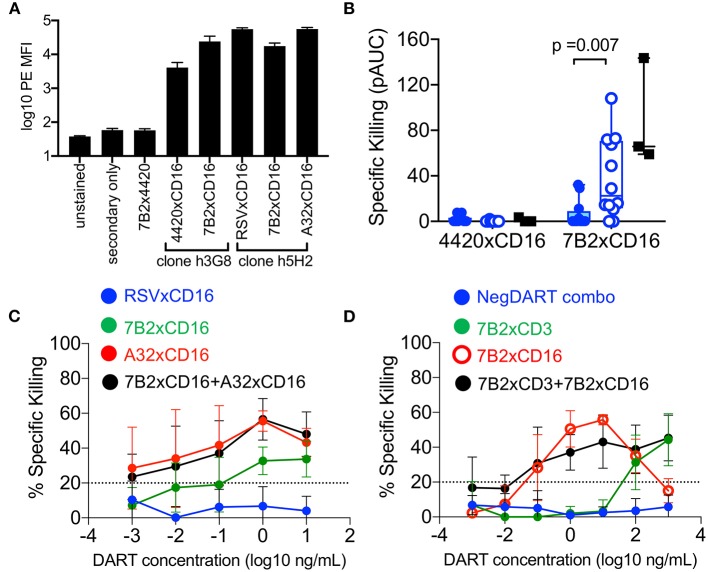
**(A)** DART-molecules with CD16-targeting arms bind to the surface of human NK cells (from *n* = 4 donors) by flow cytometry analysis. Data are reported as mean fluorescent intensities (MFI), error bars represent standard deviation. **(B)** Redirection of cord blood mononuclear cell (CBMC)-derived NK cells (from *n* = 12 CBMC samples, blue circles) for elimination of HIV-1 4403bmC5-infected autologous CD4^+^ T cells by 7B2 × CD16 or control (4420 × CD16) DART molecules in the absence of IL-15 (filled symbols) or after overnight incubation with 10 ng/mL IL-15 (open symbols). Results of assays performed with adult NK cells from three PBMC samples, in absence of IL-15, are included for comparison (black filled squares). Data are positive area under the dilution curve (pAUC) from 24 h killing assays with a NK cell to CD4^+^ T cell ratio of 5:1. **(C)** Similar levels of HIV x CD16 DART molecule-redirected killing of autologous HIV-1 4403bmC5-infeced CD4^+^ T cells were observed in assays performed with A32 × CD16 DART molecules and A32 × CD16 DART molecules combined with 7B2 × CD16 DART molecules (*n* = 3 CBMC-derived, IL-15 treated, NK cell samples, assayed in duplicate). **(D)** Redirection of CBMC (*n* = 2 samples, assayed in duplicate) for elimination of HIV-1 4403bmC5-infected autologous CD4^+^ T cells by 7B2 x CD3 DART molecules, 7B2 × CD16 DART molecules, or 7B2 x CD3 and 7B2 x CD16 DART molecules used in combination. Twenty four hours killing assay with an IL-15 treated whole CBMC to CD4^+^ T cell ratio of 30:1. Data in **(C,D)** represent mean +/– standard deviation.

To recruit and redirect NK cells, DART-molecules comprised of our previously characterized HIV-1 targeting arms (18) linked to CD16 targeting arms were developed. We used flow cytometry staining to confirm that DART-molecules with CD16-targeting arms were capable of binding to the surface of human NK cells (Figure 7A). We next evaluated the ability of cord blood NK cells to eliminate autologous CD4^+^ T cells infected with HIV-1 4403bmC5 virus when redirected by HIV × CD16 (clone hG38) DART molecules based on the 7B2 mAb. Similar to the data observed for cord blood CD8^+^ T cells, we found that resting cord blood NK cells had modest ability to eliminate autologous HIV-1 infected T cells when redirected by the 7B2 × CD16 DART molecule (Figure 7B, filled symbols). However, overnight treatment with IL-15 significantly increased (*p* = 0.007, Wilcoxon rank sum test) the cytotoxic activity of cord blood NK cells when redirected by the HIV-specific DART molecule (Figure 7B, open symbols). The 7B2 × CD16 DART molecule redirected cytolytic activity of IL-15 treated CBMC NK cells was similar to that observed in assays performed with NK cells from adult PBMC (Figure 7B, black squares). These data demonstrate that NK cells in cord blood have potential to be effective mediators of DART molecule-redirected killing after priming with IL-15. We next explored whether combinations of HIV × CD16 DART molecules specific for different epitopes improved elimination of HIV-infected cells. For these experiments, we used HIV × CD16 DART molecules (A32 × CD16 and 7B2 × CD16) and a control molecule (RSV × CD16) that were generated using a different anti-CD16 arm (clone h5H2, Figure 7A). Assays were performed using NK cells from three cord blood samples that we identified as having cytolytic activity in previous assays. Using equivalent concentrations of the A32 × CD16 and 7B2 × CD16 molecules we found that activity of the combination was similar to that of the A32 × CD16 molecule alone (Figure 7C). Thus, there was no evidence in these assays of an additive effect, nor of interference, when using these two HIV × CD16 DART molecules to mediate redirected killing with cord blood NK cells.

Finally, we investigated whether combinations of HIV × CD3 and HIV × CD16 DART molecules could work cooperatively for improved killing. To test this, we utilized IL-15 treated CBMC from two donors which we identified as having T cells and NK cells that supported redirected killing of autologous HIV-infected CD4^+^ T cells. 7B2 × CD3 and 7B2 × CD16 DART-molecules were used either alone, or in combination at equivalent concentrations. RSV × CD3 and 4420 × CD16 DART molecules in equivalent combination were used as a negative control. As shown in Figure 7D, combinations of 7B2 × CD3 and 7B2 × CD16 DART molecules did not improve the killing of HIV-1-infected autologous CD4^+^ T cells by cord blood cells in these assays.

## Discussion

Mother-to-child transmission is the principal cause of the approximate 400 new pediatric HIV-1 infections that occur each day, mostly in resource limited countries (4). Infants born into these unfortunate circumstances must strictly adhere to a lifetime of ART to control virus replication and prevent disease progression. Thus, there is a critical need for innovative therapeutic approaches enabling long-term remission or cure of infant HIV-1 infection in order to offer these children the chance to lead normal, healthy lives. One potential strategy is the use of antibody-based immunotherapy in combination with ART and latency reversing agents as a “shock and kill” approach to eliminate the virus reservoir (15, 16, 47, 48). Infants are potentially ideal candidates for immunotherapy due to the ability to initiate therapy soon after the perinatal transmission event and prior to establishment of a large virus reservoir (49, 50), and proven history of safe administration of passive antibody therapy against another infectious disease, respiratory syncytial virus (51). Here, we used human umbilical cord blood as a surrogate model of neonatal peripheral blood to test the hypothesis that HIV × CD3 DART molecules based on HIV-specific mAbs could redirect neonatal effector cells for elimination of cells infected with HIV-1. Importantly, our *in vitro* assays used autologous cord blood-derived CD4^+^ T cells as target cells, after infecting with an HIV IMC molecular clone virus encoding an HIV-1 subtype C *env* gene sequenced from plasma of a postnatally-infected infant, and identical to an isolate found in the matched maternal breast milk (34). Thus, these assays attempt to recapitulate the effector cells and HIV-1 Env-expressing target cells expected to be present in perinatally infected infants. Our results demonstrated that T cells present in cord blood can be recruited and redirected by HIV × CD3 DART molecules to eliminate HIV-1 infected target cells. This key finding supports the continued development of HIV-specific DART molecules as immunotherapies to combat pediatric HIV infection. However, we also found that the DART molecule-dependent killing activity by T cells from cord blood is significantly lessened as compared to that by T cells from adult PBMC. We therefore explored approaches to improve the cytotoxicity of cord blood effector cells with the goal of identifying strategies to maximize the potential effectiveness of DART molecule-based immunotherapy for use in early life.

We found that cord blood T cells were largely refractory to *in vitro* cytokine treatment and co-stimulatory receptor engagement intended to increase their cytolytic activity. We observed no impact of exogenous IL-12 (41), CD28 and CD137 co-stimulatory receptor engagement via specific antibodies, nor of combined use of IL-12 and co-stimulatory receptor engagement on the ability of cord blood T cells to eliminate HIV-1 infected cells when redirected by HIV × CD3 DART molecules. However, DART molecule-mediated cytolytic activity was increased when cord blood T cells were activated by TCR stimulation in the presence of IL-2. This result suggests that neonatal T cells have the capability to be potent cytolytic effectors if given sufficient activation signals. We also found that cells transitioned *in vitro* from an activated state back to a resting state had similar cytolytic activity to cells that had not experienced activation, suggesting that transient activation did not result in a durable change in the cytolytic potential of cord blood-derived CD8^+^ T cells. Comparative studies using cells from adult peripheral blood demonstrated that HIV × CD3 DART molecules effectively recruit and redirect memory and effector T cells —populations of cells that are poorly represented in cord blood. Thus, the limited maturation of T cell subsets in cord blood likely explains the observed reduced activity when compared to adult immune cells. However, it is important to consider that our *in vitro* cord blood-based experiments fail to fully model the diversity of T cell activation states and functional subsets present in an infant *in vivo*. Several lines of evidence suggest that T cells from pediatric peripheral blood may be more phenotypically and functionally diverse than those from cord blood. First, maternal and/or perinatal infant infections with cytomegalovirus and *Trypanosoma cruzi*, have been found to promote T cell maturation *in utero* and during infancy (52, 53). As cytomegalovirus, *T. cruzi*, and a myriad of other infectious agents and parasites are endemic in regions of high HIV-1 prevalence (54), it is likely that infants infected with HIV-1 via mother-to-child transmission have circulating T cells that differ in maturation, activation, and functionality when compared to those in cord blood from healthy United States-based mothers, as utilized in this study. It also has been shown that there are differences between pediatric circulating T cells, as modeled by cord blood in this study, and T cells present in pediatric tissues. Seminal work by Thome and collaborators using tissues obtained from pediatric organ donors from 2 months to 2 years of age demonstrated that, although naïve cells constitute the majority of T cells in pediatric peripheral blood, spleen, and lymph nodes, T_EM_ cells comprise a large portion of the T cells in intestinal mucosal tissues (40). Moreover, they demonstrated that memory T cells from the intestine were capable of rapid secretion of the effector cytokine IFNγ in response to stimulation. Because we found that T_EM_ cells are preferentially recruited by HIV × CD3 DART molecules, the cytolytic activity of these molecules mediated by tissue-derived T cells at mucosal sites may be higher than that observed by cord blood-derived T cells *in vitro*. It is important to note that Thome and collaborators also identified higher frequencies of T regulatory cells in pediatric blood and tissues relative to adults (40). The CD4^+^ T regulatory cells may act to either limit the cytolytic activity of infant T cell responses, or may themselves serve as effector cells for CD3 DART molecule-mediated lysis of target cells as has been previously shown (55). Thus, accurate characterization of the cytolytic activity of pediatric tissue-resident T cells when recruited by HIV × CD3 DARTs will likely require *in vivo* testing using a biologically relevant model, such as infant rhesus macaques. Finally, immune system development during early life will likely also affect the therapeutic potential of HIV × CD3 DART molecules. In fact, CD8^+^ T cell responses may mature rather quickly, as young children (2–3 years of age) have been shown to have CD8^+^ T cells with potent antiviral activity (56, 57). These observations suggest that the ability of HIV × CD3 DART molecules to mediate elimination of HIV-1-infected cells may improve over the first few years of life. Interestingly, we also found that cord blood CD4^+^ T cells contributed to elimination of infected cells *in vitro*. Whether these cells would contribute to HIV × CD3 DART molecule-dependent elimination of infected cells *in vivo* is not yet known. Additional studies using blood samples collected longitudinally from HIV-infected infants and age-appropriate non-human primate models would likely help to identify relevant populations of effector cells, and to determine the optimal timing post birth for usage of HIV × CD3 DART molecules to eliminate the HIV-1 infected cell reservoir during early life. However, the presence of higher birth levels of memory T cells, and rapid expansion of T cell memory in rhesus macaques may complicate human translation of such studies (58).

Effective control and/or cure of pediatric HIV will likely require early and aggressive interventions. Thus, the likelihood of success with the HIV-specific DART molecule-based approach might be increased by recruitment of additional populations of cytolytic effector cells to further promote eradication of infected cells, and to potentially reach additional tissues and anatomical compartments. NK cells are abundant in cord blood and pediatric peripheral blood, with cytotoxic CD56^dim^CD16^+^ NK cells representing the dominant subset (39, 45, 59). We found that HIV × CD16 DART molecules were able to recruit and redirect neonatal NK cells present in cord blood for elimination of HIV-1 infected cells and killing activity could be substantially increased by treatment with IL-15. The ability of IL-15 to augment the cytolytic activity of cord blood derived NK cells is consistent with previously reported results from assays measuring cord blood NK cell natural cytotoxicity against sensitive target cells (45). Currently, the mechanisms by which IL-15 improves NK cell cytotoxicity are incompletely defined. We previously demonstrated that IL-15 treatment of NK cells from adult peripheral blood increases the levels of cytolytic effector molecules, perforin and granzyme B (32). By contrast, we found that cord blood NK cells contained more perforin, but less granzyme B, than NK cells in adult PBMC —and the levels of neither were impacted by IL-15 treatment. Thus, intracellular abundance of perforin and granzyme B does not explain how cord blood NK cells become more cytolytic after incubation with IL-15, which suggests involvement of other activatory or regulatory processes. Exogenous IL-15 was previously shown to increase cord blood NK cell expression of intracellular adhesion molecule 1 (ICAM-1) —a cell surface protein involved in cell-to-cell interactions including formation of NK cell immunologic synapses (60). However, this effect was only seen after extended culture for 7 days (61), not after a short overnight incubation as in our study. It has also been proposed that the higher frequency of cord blood NK cells expressing cell-surface inhibitory receptor NKG2A compared to NK cells from adult peripheral blood may contribute to the reduced activity of cord blood derived NK cells (62). In contrast, we have previously demonstrated that IL-15 promotes expression of the activating receptors NKG2D and NKp30 (46). Additional research will be required to define the impact of IL-15 on the multitude of cord blood NK inhibitory and activating receptors, and to determine how these signals may integrate with the CD16-mediated signaling that occurs when NK cells interact with HIV x CD16 DART molecules.

Having found that IL-15 stimulation is able to maximize the functionality of cord blood NK cells for DART molecule-redirected killing has generated another important question: are neonatal NK cells naturally primed by IL-15 *in vivo* during HIV-1 infection? IL-15 was transiently detected in the plasma during acute HIV-1 infection of adults, with a median time of first detection being 6 days after viremia reached detectable levels in peripheral blood plasma (63). Although a similar inflammatory cytokine response is expected in neonates, it has also been demonstrated that stimulated CBMC produce less IL-15 than adult peripheral blood (64). Whether or not sufficient levels of IL-15 to activate neonatal NK cells would be present at sites of infection will require additional research. If an absence of natural IL-15 is identified, alternative strategies including the administration of recombinant human IL-15, or engineered forms of IL-15 (65, 66), in combination with the HIV × CD16 DART molecules could be tested in the infant rhesus macaque model.

Although we found that combinations of 7B2 × CD3 and A32 × CD3 DART molecules did not result in improved cytolytic activity, it is important to note that these studies were intended to explore the cytolytic potential of neonatal effector cells, not to identify optimal combinations of DART molecules. We used 7B2 and A32 DART molecules as an extension of our original study (18), but additional DART molecules targeting other Env epitopes have been described (17). It is probable that combinations of DART molecules targeting both broad-binding neutralizing and non-neutralizing epitopes, could be the most effective in recognizing the HIV Env diversity that results from the genetic diversity of HIV-1, and the variety of Env conformations that may be found on the surface of infected cells *in vivo* (43, 67, 68). Research aimed at identifying optimal combinations of anti-HIV antibodies using diverse HIV isolates, and patient-derived latent reservoir virus (Tuyishime et al., submitted) will likely help identify optimal combinations of DART molecules.

At present, blinatumomab, a CD19 × CD3 antibody-based bispecific molecule, is the only example of an immune cell-engaging bispecific drug that has FDA approval for the treatment of disease—B cell acute lymphoblastic leukemia (69, 70). However, there are dozens of bispecific antibody-based molecules in various stages of development, and clinical testing, against multiple types of cancers (71, 72). This includes several DART molecules being evaluated for potential use in treatment of leukemias and lymphomas (73, 74), colorectal cancer (55), and solid tumors (75). The majority of bispecifics in development are intended to recruit T cells via an anti-CD3 arm, however NK cell recruitment by anti-CD16 targeting is also being explored (76, 77). The continued development and clinical testing of DART molecules, and other antibody-based bispecifics, for use against human cancers will provide important safety, durability, and efficacy data that will help inform the best strategies for use of these types of therapies against other diseases, including HIV-1 infection.

In summary, we demonstrated that HIV × CD3 and HIV × CD16 DART molecules can recruit and redirect cytolytic immune cells present in cord blood to eliminate autologous T cells infected with HIV-1, albeit less effectively than cells present in adult peripheral blood. Our work supports the continued development of these antibody-based molecules as components of passive immunization strategies aimed at treatment and cure of pediatric HIV. However, future studies using early life blood and tissue samples, and infant or juvenile preclinical animal models, will be required to identify tenable strategies to optimize their efficacy.

## Data Availability Statement

The datasets generated for this study are available on request to the corresponding author.

## Ethics Statement

Anonymized human umbilical cord blood donations that failed to meet the criteria required for clinical use were obtained with informed written consent. Human peripheral venous blood was collected by leukapheresis from healthy consenting adult volunteers (28, 29). All samples were collected in accordance with the policies and regulations of the Duke Health Institutional Review Board.

## Author Contributions

JP conceived the study, performed experiments, analyzed and interpreted data, and wrote the manuscript. RE performed experiments and analyzed data. SJ performed statistical analyses. TH and JAP performed experiments and analyzed data. C-YL, LL, GD, and JN led the design, development, and production of the bispecific dart molecules. SP isolated and characterized the breast milk transmitted/founder HIV-1 virus. TD collected and characterized the adult leukapheresis samples. SP and GF interpreted data and contributed to design of the study. All authors commented on the manuscript and approved the final version.

## Conflict of Interest

C-YL, LL, GD, and JN are employees of MacroGenics, Inc., and receive salaries and stock options as compensation for their employment. The remaining authors declare that the research was conducted in the absence of any commercial or financial relationships that could be construed as a potential conflict of interest.
